# HPLC-ED Analysis of Phenolic Compounds in Three Bosnian *Crataegus* Species

**DOI:** 10.3390/foods7050066

**Published:** 2018-04-24

**Authors:** Dušan Čulum, Amira Čopra-Janićijević, Danijela Vidic, Lejla Klepo, Azra Tahirović, Neđad Bašić, Milka Maksimović

**Affiliations:** 1 Department of Chemistry, Faculty of Science, University of Sarajevo, Zmaja od Bosne 33-35, 71 000 Sarajevo, Bosnia and Herzegovina; dusan_c90@hotmail.com (D.Č.); danijela.vidic@gmail.com (D.V.); klepolejla@gmail.com (L.K.); mmaksimo@pmf.unsa.ba (M.M.); 2 Department of Forest Ecology, Faculty of Forestry, University of Sarajevo, Zagrebačka 20, 71 000 Sarajevo, Bosnia and Herzegovina; atahirovic2001@yahoo.com (A.T.); basicnedzad@yahoo.com (N.B.)

**Keywords:** *Crataegus*, Soxhlet, ultrasound extraction, flavonoids, phenolic acids, HPLC-ED

## Abstract

The aim of this work was the qualitative and quantitative determination of selected phenolic compounds in three *Crataegus* species grown in Bosnia. *Crataegus* plants are consumed for medicinal purposes and as foodstuff in the form of canned fruit, jam, jelly, tea, and wine. Two samples of plant material, dry leaves with flowers, and berries of three *Crataegus* species—*Crataegus rhipidophylla* Gand., *Crataegus x subsphaericea* Gand., and *Crataegus x macrocarpa* Hegetschw.—were analyzed. Twelve ethanolic extracts were isolated from the selected plant material using Soxhlet and ultrasound extraction, respectively. Soxhlet extraction proved to be more effective than ultrasound extraction. A simple and sensitive method, high-performance liquid chromatography with electrochemical detection, HPLC-ED, was used for the simultaneous determination of phenolic acids and flavonoids in *Crataegus* species. The content of gallic acid in the extracts ranged from 0.001 to 0.082 mg/g dry weight (DW), chlorogenic acid from 0.19 to 8.70 mg/g DW, and rutin from 0.03 to 13.49 mg/g DW. Two flavonoids, vitexin and hyperoside, commonly found in chemotaxonomic investigations of *Crataegus* species, were not detected in the examined extracts. In general, leaves with flowers samples are richer in gallic acid and rutin, whereas the berries samples are richer in chlorogenic acid. Distinct similarities were found in the relative distribution of gallic acid among the three species. Extracts of *C. x macrocarpa* had the highest content of all detected compounds, while significant differences were found in rutin content, depending on the plant organ. To the best of our knowledge, this is the first study reporting content of phenolic compounds in *Crataegus rhipidophylla* Gand., *Crataegus x subsphaericea*, and *Crataegus* *x* *macrocarpa* from Bosnia.

## 1. Introduction

The genus *Crataegus* L., commonly called hawthorn, is a large genus that comprises low trees or shrubs and is widespread mainly in the native temperate regions of the Northern Hemisphere [1,2]. The subcontinental species of *Crataegus rhipidophylla* Gand. is native to the area extending from Scandinavia and the Baltic region to France, the Balkan Peninsula, Turkey to the Caucasus and the Crimea [1]. This species often crosses with other species of *hawthorn* in nature, thus forming hybrid complexes. *Crataegus x macrocarpa* Hegetschw. is the hybrid formed by crossing between *C. laevigata* (Poiret) DC. and *C. rhipidophylla*. It has long been known of and is common. This hybrid is widespread from southern Scandinavia to the Baltic region and Central Europe [1]. Also, the hybrid *Crataegus x subsphaericea* Gand. (*C. monogyna* Jacq. x *C. rhipidophylla*) spreads naturally from southern Scandinavia, the Baltic region to Central Europe, the Balkan Peninsula, Turkey, Caucasus up to the Crimea [1]. The presence of these taxa was also confirmed in Bosnia and Herzegovina [3,4].

Wild growing plants generate a lot of interest as a source of bioactive compounds and are reported to have considerable nutritional and medicinal value. Numerous studies have demonstrated that the various species of *Crataegus* possess pharmaceutical properties and, therefore, have been used in medicine to prevent or treat cardiovascular diseases [5,6,7], to improve circulation and to treat hypertension, arrhythmia, angina pectoris, and high blood cholesterol levels [8]. Antiradical activities of hawthorn extracts have been proven by numerous researches [9,10,11]. Fruits, leaves, and flowers of *Crataegus* species contain major bioactive phenolic compounds, such as phenolic acids, flavonoids, anthocyanidins [12,13,14]. Furthermore, various parts of plants belonging to *Crataegus* species, in particular dried berries, represent an important raw material in the food industry, as they are being used in production of different herbal teas, juices, and wine, as well as jams, jellies, and canned fruits [8,10,15,16,17,18].

Phenolic acids and flavonoids are the most abundant polyphenols in human diet [19,20,21]. Phenolic acids are found in plants either in free forms, as esters and glycosides, or bounded in the form of complexes. Flavonoids may be present as aglycones or glycosides, depending upon their structure [21]. Gallic acid is a derivate of benzoic acid and has antioxidant properties in the cell system and plays a neuroprotective role [22]. It has been reported that chlorogenic acid shows antioxidant, anti-inflammatory, and neuroprotective properties [23]. Rutin—one of the most abundant flavonol glycoside in plants—has been used in medicine as an antimicrobial, antifungal, and antioxidant agent, as well as in the treatment of chronic diseases such as diabetes, cancer, and hypertension [24]. Generally, flavonoids and phenolic acids are recognized as the most important plant antioxidants [25]. 

Since phenolic compounds are the main bioactive components found in leaves, flowers, and fruits of different official medicinal hawthorn species [26], the aim of this work was to determine phenolic acids and the flavonoid pattern responsible for chemotaxonomic markers, differentiating species and varieties. To the best of our knowledge, this is the first study reporting phenolic compounds in the *Crataegus rhipidophylla* Gand., *Crataegus x subsphaericea*, and *Crataegus x macrocarpa* from Bosnia.

## 2. Materials and Methods

### 2.1. Plant Material

The plant material of *Crataegus rhipidophylla*, *Crataegus x subsphaericea*, and *Crataegus x macrocarpa* were collected in June (leaves with flowers, LF) and September (berries, B) on mountain Trebević, near Sarajevo, Bosnia and Herzegovina. The taxonomic identification of these three autochthonous *Crataegus* species was performed by a comparative morphological analysis within a sympatric population. In order to avoid the influence of pedoclimatic conditions, all analyzed samples were collected from the same area. Six samples were collected, three samples of leaves with flowers and three samples of berries, with an approximate mass of 300 g per sample. Samples for extractions were prepared using the quartering method of composite samples.

Plant material was air-dried at ambient temperature in a ventilated room to a constant weight. After drying, the samples were stored in paper bags, in a dry place, until use. The identification of the plant material was confirmed by a plant taxonomist Bašić Neđad, and the voucher specimens have been kept at the herbarium of the Department of Forest Ecology at the Faculty of Forestry, of the University of Sarajevo.

### 2.2. Chemicals 

All employed standards, reagents, and solvents were of the highest purity available and purchased from Sigma-Aldrich Co. (Darmstadt, Germany), except the chlorogenic acid and hyperoside that were purchased from Acros Organics, Gell, Belgium and HWI ANALYTIK GmbH, Rülzheim, Germany respectively.

The stock solutions of standards, at a concentration of 1 mg/mL, were prepared by dissolving standard compounds in HPLC-grade methanol.

### 2.3. Extraction

Extracts of leaves with flowers samples and berries samples were prepared by Soxhlet (S) and ultrasound extraction (US) using 96% ethanol as a solvent [27,28,29]. The Soxhlet extractor was used for extraction of 20 g of dried plant material in 500 mL of solvent for 5 h. Ultrasound extraction was performed using an ultrasonic water bath BRANSON-Smith Kline Company, Oxnard, CA, USA B-32 (150 W, 240 V, 50–60 Hz) for 1.5 h, extracting 10 g of plant sample in 100 mL of solvent. After extraction, the solvent was removed using a rotary vacuum evaporator and dry extracts were stored in the refrigerator at 4 °C until use. HPLC-ED (Shimadzu LCSOL Single-LCEN, Kyoto, Japan) was employed for qualitative and quantitative analysis of phenolic acids and flavonoids.

### 2.4. HPLC Analysis

The HPLC-ED conditions were as follows: potential range and flow rate were 840 mV and 1 mL/min, respectively. The chromatographic separation was performed on a Hypersil ODS column Hewlett Packard, Waldbronn, Germany (5 µm 200 × 4.6 mm). Methanol–acetonitrile–water–glacial acetic acid (20:10:70:1) was used as the mobile phase. The injected volume was 20 µL, 0.02 Hz filter, range of 50 nA, and the column was thermostatted at a temperature of 25 °C [30]. The results were expressed per gram of dry weight (DW) of plant material.

### 2.5. Statistical Analysis

All measurements were carried out in triplicate experiments and the results are expressed as mean value ± standard deviations. The data were subjected to one-way analysis of variance (ANOVA) and the means were separated by Duncan multiple range test (IBM SPSS Statistics version 20, IBM Corp., Armonk, NY, USA). Differences were considered significant at *p* < 0.05. 

## 3. Results and Discussion

### 3.1. Extraction Yield

Comparing all three used plant species, Soxhlet extracts of leaves with flowers samples of *C. rhipidophylla* (26.8%) and *C. x macrocarpa* (26.7%) obtained almost the same yield. The lowest yield was obtained from berries sample of *C. x macrocarpa* using ultrasonic extraction (0.81%). Soxhlet extraction proved to be more efficient for all samples and for all plants analyzed. The yields for leaves with flowers samples were three times higher than for berries samples (Table 1). 

### 3.2. Identification and Quantification

**Figure 1:**
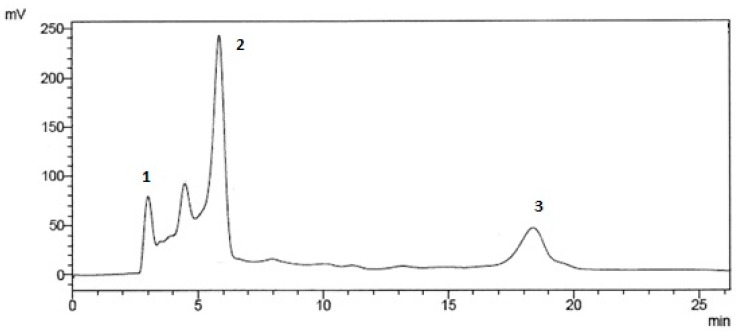
HPLC chromatogram of the extract of *C. rhipidophylla* berries sample (B) extracted by Soxhlet extraction S; 1: gallic acid (Rt = 3.7 min); 2: chlorogenic acid (Rt = 7.2min); 3: rutin (Rt = 18.5 min).

The content of phenolic compounds in the extracts was analyzed using an HPLC system with electrochemical detection. An example of an HPLC chromatogram of *C. rhipidophylla* extract of berries sample extracted using Soxhlet extraction is given below (Figure 1).

Gallic and chlorogenic acids were found in all analyzed extracts. Rutin was also detected in all extracts, except in berries extracts of the *C. x macrocarpa* obtained by both types of extraction (Table 2). Two flavonoids, vitexin and hyperoside, commonly found and used as chemotaxonomic markers of *Crataegus* species were not detected in the samples.

The content of rutin in the tested samples ranged from 0.03 for *C. x subsphaericea* B (US) to 13.49 mg/g DW for *C. x macrocarpa* LF (S); gallic acid from 0.001 for *C. x macrocarpa* B (US) to 0.082 mg/g DW for *C. x macrocarpa* LF (S); and chlorogenic acid varied ranging from 0.19 for *C. x macrocarpa* B (US) to 8.70 mg/g DW *C. x macrocarpa* B (S).

It is important to note that the content of rutin was significantly higher in *C. x macrocarpa* (LF) extracted by Soxhlet extraction, whilst in the berries samples rutin was not detected at all. The content of gallic acid in all the extracts was considerably lower than the content of chlorogenic acid and rutin. *Crataegus x macrocarpa* had the highest content of rutin and gallic acid in (LF) as well as chlorogenic acid in (B) when prepared using the Soxhlet extraction. In general terms, leaves with flowers samples were richer in the content of gallic acid and rutin, while the berries samples contained a higher amount of chlorogenic acid.

Thus, when comparing our results obtained in relation to the analyzed components, it can be observed that phenolic profile of hybrid *C. x macrocarpa* is more similar to the original *Crataegus rhipidophylla* than the hybrid *C. x subsphaericea.*

There is no apparent correlation between the type of extraction and the content of the analyzed compounds.

Significant differences (*p* < 0.05) between extracts have been determined for the content of rutin, gallic acid, and chlorogenic acid with analysis of variance. Means for groups in homogeneous subsets are displayed in Table 2. The results of Duncan test confirm a notable number of significant differences between the extracts within species and between species for all analytes. Seven subsets were obtained for rutin and gallic acid, while five homogeneous subsets were obtained for chlorogenic acid. 

In conclusion, these preliminary results indicate that there are significant differences in the content of rutin, gallic acid, and chlorogenic acid in leaves with flowers samples and berries samples both within species and between species. 

The results obtained for the content of rutin in three Bosnian *Crataegus* species are in accordance with the literature data, in which fruits have much lower content of rutin than leaves and flowers [7,31]. Ringl et al. [32] reported the content of flavonoids in the flowers of *C. x macrocarpa*, *C. rhipidophylla* and *C. leavigata,* from Austria and Germany. In the 11 samples of *C. x macrocarpa* analyzed by Ringl, rutin was detected in a significantly lower concentration (0.9–1.6 mg/g DW) than in our study. The content of vitexin was found in a concentration, ranging from 0.1 to 0.6 mg/g DW. Hyperoside was found in 10 samples (0.1–0.9 mg/g DW), whilst it was not detected in 1 of the analyzed samples, which is consistent with the results obtained in our study. In the extract of *C. rhipidophylla*, rutin was detected at a considerably lower concentration (0.4 mg/g DW) than in our study (1.23–8.21 mg/g DW). Vitexin and hyperoside were detected at a concentration of 1 mg/g and 5.5 mg/g DW, respectively. Isovitexin was not detected in any of *C. x macrocarpa* samples, whilst it has been found in *C. rhipidophylla*. In the samples of *C. leavigata*, vitexin, isovitexin, and rutin were not detected. The content of vitexin and vitexin O-rhamnoside [7] in the flowers of *C. rhipidophylla* var. *rhipidophylla* were 0.1 mg/g DW and 4.1 mg/g DW, whereas in *C. x macrocarpa* they were 0.1–0.6 mg/g DW and 1.4–6.4 mg/g DW. Vitexin was not found in the flowers of *C. leavigata* and *C. microphylla* nor in the leaves of *C. scabrifolia*, as was the case in our *Crataegus* samples.

To the best of our knowledge, there is no available data relating to the content of phenolic acids in *Crataegus* species used in this study, however, in some other *Crataegus* spp., chlorogenic and gallic acid, as well as procyanidins, flavonols, and C-glycosylflavons were detected [6,33].

## 4. Conclusions

The content of the phenolic compounds in the *Crataegus* extracts is rather high and can serve as a good source of bioactive compounds for medicinal purposes and as a foodstuff. *Crataegus x macrocarpa* had the highest content of rutin and analyzed phenolic acids. 

Our preliminary results indicate significant differences in the content of rutin, gallic acid, and chlorogenic acid within the species itself and between species. To the best of our knowledge, this is the first study reporting on the content of phenolic compounds in the *Crataegus rhipidophylla* Gand, *Crataegus x subsphaericea*, and *Crataegus x macrocarpa* from Bosnia.

## Figures and Tables

**Table 1:** Extract yield of the *Crataegus* species.

Samples	Extraction	Yield (%)
*C. x subsphaericea* (B)	Soxhlet	2.5
*C. x subsphaericea* (B)	Ultrasound	1.5
*C. x subsphaericea* (LF)	Soxhlet	23.9
*C. x subsphaericea* (LF)	Ultrasound	7.7
*C. rhipidophylla* (B)	Soxhlet	6.5
*C. rhipidophylla* (B)	Ultrasound	1.2
*C. rhipidophylla* (LF)	Soxhlet	**26.8**
*C. rhipidophylla* (LF)	Ultrasound	10.0
*C. x macrocarpa* (B)	Soxhlet	8.7
*C. x macrocarpa* (B)	Ultrasound	0.8
*C. x macrocarpa* (LF)	Soxhlet	**26.7**
*C. x macrocarpa* (LF)	Ultrasound	8.9

LF: leaves with flowers; B: berries; The highest yield in **bold**.

**Table 2:** The content of rutin and gallic and chlorogenic acid in the *Crataegus* samples.

Plant	Samples	Rutin	Gallic Acid	Chlorogenic Acid
		mg/g DW	mg/g DW	mg/g DW
*C. x subsphaericea*	B (S)	0.22 ± 0.02 ^a^	0.009 ± 0.001 ^b^	1.76 ± 0.09 ^b^
	B (US)	0.03 ± 0.01 ^a^	0.001 ± 0.000 ^a^	0.35 ± 0.02 ^a^
	LF (S)	0.31 ± 0.01 ^a^	0.002 ± 0.000 ^a^	0.22 ± 0.01 ^a^
	LF (US)	3.95 ± 0.34 ^e^	0.018 ± 0.002 ^c^	1.79 ± 0.04 ^b^
*C. rhipidophylla*	B (S)	1.73 ± 0.03 ^c^	0.043 ± 0.002 ^e^	2.84 ± 0.02 ^c^
	B (US)	1.23 ± 0.02 ^b^	0.024 ± 0.001 ^d^	3.99 ± 0.26 ^d^
	LF (S)	2.71 ± 0.02 ^d^	0.068 ± 0.009 ^f^	2.76 ± 0.12 ^c^
	LF (US)	8.21 ± 0.48 ^f^	0.066 ± 0.003 ^f^	2.63 ± 0.19 ^c^
*C. x macrocarpa*	B (S)	nd	0.017 ± 0.001 ^c^	8.70 ± 0.59 ^e^
	B (US)	nd	0.001 ± 0.000 ^a^	0.19 ± 0.01 ^a^
	LF (S)	13.49 ± 0.00 ^g^	0.082 ± 0.001 ^g^	2.97 ± 0.02 ^c^
	LF (US)	4.22 ± 0.01 ^e^	0.011 ± 0.000 ^b^	1.45 ± 0.09 ^b^
LOD (mg/mL)		0.0006	0.00003	0.0002
LOQ (mg/mL)		0.002	0.0001	0.0006

B: berries, LF: leaves with flowers, US: ultrasound; S: Soxhlet; DW: dry weight; nd: not detected. Values are presented as mean ± standard deviation (*n* = 3). Values in the same column with different letters in superscript are significantly different at *p* < 0.05. LOD: limit of detection and LOQ: limit of quantification of detected compounds.
